# Density clustering-based automatic anatomical section recognition in colonoscopy video using deep learning

**DOI:** 10.1038/s41598-023-51056-6

**Published:** 2024-01-09

**Authors:** Byeong Soo Kim, Minwoo Cho, Goh Eun Chung, Jooyoung Lee, Hae Yeon Kang, Dan Yoon, Woo Sang Cho, Jung Chan Lee, Jung Ho Bae, Hyoun-Joong Kong, Sungwan Kim

**Affiliations:** 1 Interdisciplinary Program in Bioengineering, Graduate School, Seoul National University, Seoul, 08826 Korea; 2 Innovative Medical Technology Research Institute, Seoul National University Hospital, Seoul, 03080 Korea; 3 Department of Transdisciplinary Medicine, Seoul National University Hospital, Seoul, 03080 Korea; 4 Department of Medicine, Seoul National University College of Medicine, Seoul, 03080 Korea; 5 Department of Internal Medicine and Healthcare Research Institute, Healthcare System Gangnam Center, Seoul National University Hospital, Seoul, 06236 Korea; 6 Department of Biomedical Engineering, Seoul National University College of Medicine, Seoul, 03080 Korea; 7 Institute of Bioengineering, Seoul National University, Seoul, 08826 Republic of Korea; 8 Institute of Medical and Biological Engineering, Medical Research Center, Seoul National University, Seoul, 03080 Korea; 9 Artificial Intelligence Institute, Seoul National University, Research Park Building 942, 2 Fl., Seoul, 08826 Korea; 10 Medical Big Data Research Center, Seoul National University College of Medicine, Seoul, 03087 Korea

**Keywords:** Colonoscopy, Biomedical engineering

## Abstract

Recognizing anatomical sections during colonoscopy is crucial for diagnosing colonic diseases and generating accurate reports. While recent studies have endeavored to identify anatomical regions of the colon using deep learning, the deformable anatomical characteristics of the colon pose challenges for establishing a reliable localization system. This study presents a system utilizing 100 colonoscopy videos, combining density clustering and deep learning. Cascaded CNN models are employed to estimate the appendix orifice (AO), flexures, and "outside of the body," sequentially. Subsequently, DBSCAN algorithm is applied to identify anatomical sections. Clustering-based analysis integrates clinical knowledge and context based on the anatomical section within the model. We address challenges posed by colonoscopy images through non-informative removal preprocessing. The image data is labeled by clinicians, and the system deduces section correspondence stochastically. The model categorizes the colon into three sections: right (cecum and ascending colon), middle (transverse colon), and left (descending colon, sigmoid colon, rectum). We estimated the appearance time of anatomical boundaries with an average error of 6.31 s for AO, 9.79 s for HF, 27.69 s for SF, and 3.26 s for outside of the body. The proposed method can facilitate future advancements towards AI-based automatic reporting, offering time-saving efficacy and standardization.

## Introduction

Colonoscopy serves as the primary screening method for identifying neoplastic diseases (such as colon polyps and cancer) and inflammatory conditions (like Crohn’s disease and ulcerative colitis) within the colon^1,2^. However, the efficacy of colonoscopy is hindered by inter-observer and intra-observer variability influenced by the experience and expertise of the endoscopist^3−6^.

To address these pitfalls, artificial intelligence (AI) has been introduced to gastrointestinal (GI) endoscopy, aiming to enhance procedure effectiveness and mitigate human errors. Numerous studies have demonstrated that computer-assisted detection and diagnosis systems can significantly improve an endoscopist’s ability to detect and optically diagnose colon polyps^7−9^. Commercial products like GI Genius™ (Medtronic, Minneapolis) and CAD EYE™ (Fujifilm Holding Corporation, Tokyo, Japan) have been introduced for clinical use. AI technologies in colonoscopy are advancing towards autonomously assessing quality indicators, such as cecal intubation rates, bowel preparation scores, and the generation of procedural reports^10,11^.

Following the inspection, endoscopists are required to produce high-quality reports detailing bowel preparation by colon sections and specifying disease phenotypes, including lesion location or extent^10,11^. This information is crucial for clinical decision-making regarding accurate diagnosis and treatment plans^12−14^. Additionally, knowledge of the colon’s location aids in revisiting polyps during subsequent insertions, especially if they are too large or complicated to resect during the initial inspection, or necessitate transfer to a more advanced medical facility for surgical intervention, the colon location provides supplementary information to revisit the polyps at subsequent insertion^15,16^. Such AI systems have the potential to enhance procedure quality and accuracy while reducing the workload on medical professionals^10,11^.

The colon, comprising six segments (cecum, A-colon, T-colon, D-colon, S-colon, and rectum), is a deformable organ, posing a challenge to developing an AI model that recognizes its anatomical regions^17,18^. Some studies have delved into analyzing the motion vector in scope images, calculating scope movement^19^, and classifying the location of the cecum or flexure^11,20^. Bao et al.^21^ introduced a method incorporating an RF sensor inside a capsule endoscope, tracking its position through an external receiver. Armi et al.^22^ generated videos by capturing simulator footage to determine the camera pose in colonoscopy images. They employed a CNN model trained on this simulated data to estimate the camera’s intrinsic parameters, thus approaching the localization of the colonoscope camera. Laiz et al.^23^ developed an algorithm distinguishing between internal and external regions, including the entrance and exit of the colon. They utilized information visualizing the camera’s motion along with temporal data for this development. In summary, recognizing the colon’s positional segments allows for tailored diagnosis and treatment when diseases are detected, enhancing surgery efficiency, facilitating postoperative tracking and monitoring, and enabling effective medical support specific to each region. However, the location segment information needed to be more sufficient and accurate in those studies. Additionally, estimating the camera’s trajectory through motion is only feasible when translation and rotation transformation information is reliably captured.

This study focuses on developing a model capable of identifying anatomical sections of the colon through density clustering, considering anatomical continuity. Notably, the training data for the model were meticulously chosen with the consensus of eight gastroenterologists, comprising information from 100 patients, including 4127 images related to the cecum area and an additional set of 5546 images for flexure detection.

During the anatomical section clustering phase, each image was categorized as non-informative or informative, as illustrated in Supplementary Figure A1. Automatic non-informative filtering assigned labels to images as Appendix orifice, flexure, or outside of the body. This approach allows the model to autonomously categorize the colon into three main sections: right colon, middle colon, and left colon. Importantly, this novel approach eliminates the need for estimating the camera’s trajectory, avoids camera-specific parameters, and dispenses with the requirement for 3D reconstruction. It stands out as a distinctive methodology by classifying colon segments directly from the original colonoscopy image, requiring no additional components or separate procedures.

## Material and methods

### Dataset

Due to the colon’s deformable nature, precise localization poses a challenge for endoscopists during colonoscopy. They rely on a combination of factors, including markers for insertion depth, the shape of the lumen, and the presence of adjacent organs such as the liver, gallbladder, and spleen. The colon, with its six segments—cecum, ascending colon (A-colon), transverse colon (T-colon), descending colon (D-colon), sigmoid colon (S-colon), and rectum—is typically divided into three main sections: the right colon (comprising the cecum and A-colon), the middle colon (T-colon), and the left colon (D-colon, S-colon, rectum). Notably, the hepatic flexure (HF) marks the boundary between the right and middle colon and features the liver and gallbladder, appearing as a distinctive blue spot resembling a half-moon shape in colonoscopy. Similarly, the splenic flexure (SF) between the middle and left colon reveals the presence of the spleen as a distinct blue spot^18^. Our system, designed to leverage anatomical features such as flexures, consists of three layers that progressively incorporate clinical information. During 100 colonoscopy procedures performed using the EVIS LUCERA ELITE Video Colonoscope CF-HQ290 (Olympus Corporation, Tokyo, Japan), six experienced gastroenterologists (excluding GEC and JHB) meticulously annotated colon segments with timestamps for key landmarks, including the appendiceal orifice (AO), HF, SF, sigmoid/descending colon junction (SDJ), and recto/sigmoid junction (RSJ). Real-time monitoring of insertion depth was facilitated through the colonoscopy’s marked length scale. These annotations underwent cross-verification by another observer (GEC), and a third gastroenterologist (JHB) conducted a thorough review of the 100 video clips to ensure accuracy. All participating physicians were seasoned experts in the field of endoscopy. Since we used retrospective data, we waived the need to obtain informed consent. The retrospective data were collected from the previous study’s database, and informed consent from the patients was waived^24^. The study adhered to ethical guidelines and received approval from the Seoul National University Hospital Institutional Review Board (IRB number H-2001-083-1095), following the Declaration of Helsinki. Videos were recorded at a resolution of 720 × 480 pixels, at 30 frames per second, using H.264/MPEG-4 (Part10) AVC compression. From the 100 video clips, still images were extracted at a rate of three frames per second (resulting in PNG files captured every 0.3 s using VirtualDub software)^11,25,26^. Demographic data is provided in Table 1. We uniformly cropped patient information in image preprocessing. The final training image size was 520 × 410 pixels. In this study, we established two criteria, ‘out of focus’ and ‘specularity,’ to define non-informative images. ‘Out of focus’ refers to cases involving blurring or motion blur, impairing visibility, while ‘specularity’ pertains to instances where light reflection causes pixel saturation. Informative images eligible for input into the model in endoscopic footage exclude those falling into these two scenarios (Supplementary Figure A1 provides detailed examples). The "removal of non-informative frames" process utilized the global contrast factor (GCF)^27−29^ as the threshold to exclude out-of-focus images and employed the specular reflection detection method^30^ to eliminate specularities^31^.

**Table 1:** Patient demographics.

Characteristics	Total (N = 100)
Age (years)	
Median	53
Range	27–78
Gender	
Male	42
Female	58
Inspection time (in withdrawal phase from cecum to anus)	
Median	7 min 53 s
Range	3 min 34 s–27 min 37 s
Number of polyps	
Average	0.48
Range	0–1

### Development of the anatomical section recognition algorithm

**Figure 1:**
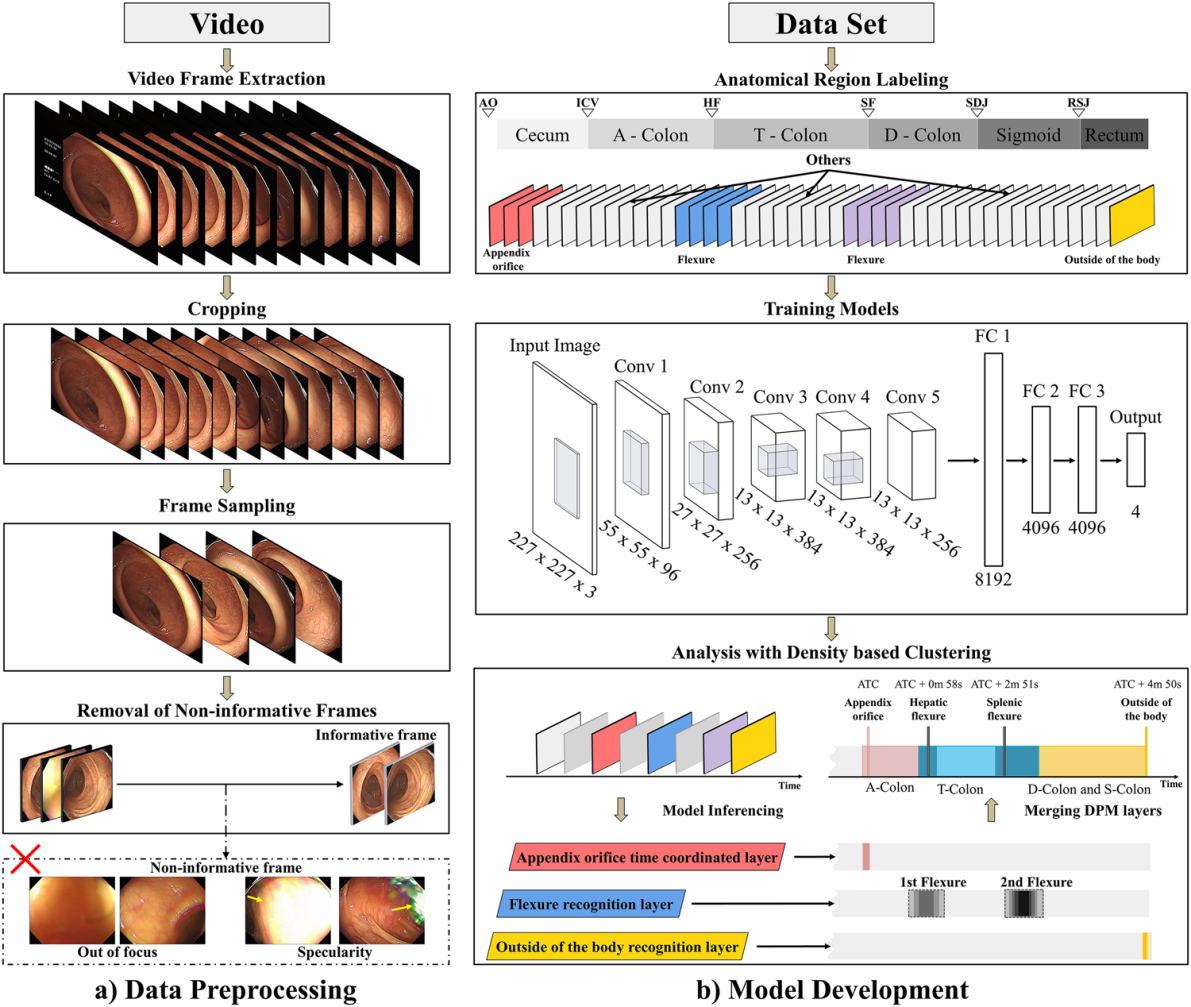
Overall system of the density clustering-based automatic reporting system. (**a**) The Data Preprocessing phase involves cropping frames extracted from videos, sampling one frame per three, and removing non-informative frames. (**b**) The Model Development section encompasses the training process of the model and the analytical process of distinguishing colon segments through the model. ATC: appendix orifice time coordinated.

Figure 1 depicts a comprehensive pipeline for the recognition of anatomical sections. The process consists of two main components: data and model. During data preprocessing, frames are extracted from a 30fps video, and only relevant regions for endoscopic training are cropped. To address the computational demand, frames are sampled at three frames per second. The final preprocessing step involves experimentally defining (see supplementary material A) and removing non-informative frames based on criteria for out-of-focus and specularity images. In the model development phase, the remaining informative frames are annotated, used for training, and the resulting model is applied for analysis.

The primary objective of this study is to improve the macroscopic visualization of the lower gastrointestinal (GI) tract, specifically the colon, which presents challenges due to its deformable properties. To address this difficulty, we utilize time-series data from endoscopic videos, allowing for a segment-by-segment macroscopic view. The system is designed with three distinct layers: The Appendix Orifice Time-Coordinated (ATC) layer, the Flexure Recognition layer, and the "Outside of the Body" Recognition layer.

We employed the modified AlexNet algorithm^32^ (refer to supplementary material B) with a training batch size of 8 incorporating a decay rate of 1e-4 and a learning rate of 1e-3. The loss function used was categorical cross-entropy, and the optimization was carried out using the Adam optimizer. We compared various CNN architectures, including EfficientNet, Inception, SE-ResNet, and DenseNet, as potential models for DPM layers. Through this evaluation, we found that a modified version of AlexNet performed the best for utilization in DPM layers. The performance comparison table is presented in Supplementary Table B1.

The modified AlexNet’s architecture entailed replacing the activation function of the original AlexNet from ReLU to Swish and iteratively applying BatchNormalization, Activation, and Maxpooling at each layer. Additionally, we increased the number of Flatten Layers from two to three in the original AlexNet, aiming to achieve a deeper layer structure. We utilized this modified AlexNet in our study and trained it using the aforementioned parameters, undergoing 86 epochs for the ATC model, 96 epochs for flexure classification, and 17 epochs for Outside of the body classification. This variation across models was due to the application of early stopping.

Our hardware setup comprised two NVIDIA® GeForce® RTX 2080 Ti 12 GB GPUs, an Intel® Core™ i7-9700 K CPU @ 3.60 GHz, and 128 GB RAM. Significantly, anatomical segmentation of the large intestine considered its irregular shape and variability, utilizing density-based spatial clustering of applications with noise (DBSCAN)^33^. After applying DBSCAN, results were represented through kernel density estimation (KDE) graphs. DBSCAN was further applied to all three decision graphs to obtain clusters, and the density-based potential map (DPM) visualized these clusters as KDE graphs.

#### Density-based potential map (DPM)

During a colonoscopy inspection, the output image captured by the scope exhibits dynamic movements due to rotation, shaking, and reciprocation as it navigates through each segment. Consequently, obtaining a clear view of informative features, even when the scope is positioned within a specific anatomical region, becomes a challenging endeavor. Significantly, pinpointing the precise detection time requires considerable effort. Moreover, due to the colon’s anatomical structure, the colonoscope continually examines the lumen during the withdrawal phase. This results in each segment being observed multiple times within a short timeframe, without a single timestamp to rely on. Consequently, defining the locations of the AO, flexures, or areas outside of the body becomes impossible. In response, we have generated a KDE graph to illustrate the model’s predictions.

(i) The execution of the DPM for predictions follows the subsequent sequence. The withdrawal phase videos from colonoscopic time-series data are processed by selecting only three frames per second and subsequently eliminating non-informative frames.
(ii) The retained frames become candidate sample points for the clustering step. A classification model trained on anatomical sections (AO, Flexures, and outside of the body) determines whether a frame qualifies as a sample point.
(iii) Frames identified as corresponding segments by the classification model (e.g., AO model classifying AO presence) serve as sample points for DBSCAN input.
(iv) Densely clustered sample points labeled AO on the video timeline pinpoint the locations of AO. Replicating this process for the Flexure and Outside of the body models and overlaying the results in the time domain allows for the classification of the entire colon into right, middle, and left sections.

We hypothesized that as the endoscope approaches specific anatomical regions during the examination, the classifier would consistently recognize frames with elevated confidence levels. Subsequently, we determined the probability of encountering the intended anatomical section by systematically analyzing the video frames. In step 2 of the aforementioned method, determining whether the frames are used as valid data points relied on the confidence score of the CNN model, with a threshold set at 0.5. This threshold aligns with the True Positive criterion commonly adopted in most deeplearning based decision making tasks^34,35^.

In this context, for each data point within a cluster, we evaluated the proximity of neighboring data points within a specified radius (epsilon) and assessed whether there were a sufficient number of neighboring points (minimum points) to satisfy a particular threshold. The neighborhood of an arbitrary point ‘$p$’ is defined as follows^36^.

1 $${N}_{Eps}=\left\{q \epsilon \frac{{\text{D}}}{dist\left(p,q\right)}<{\text{Eps}}\right\},$$

2 $${N}_{Eps}\left(p\right)> MinPts,$$

where ${\text{D}}$ is the group of data points. If the vicinity of point p contains at least MinPts, then $p$ is a core point.

We conducted an experiment to identify AO, flexure and the section outside the body. Using this dataset, we generated a potential map (refer to Fig. 3). In our study, we employed a technique known as DPM for the analysis of colonoscopy videos. This approach facilitated the segmentation of the colon into three distinct parts: the right colon, middle colon (T-colon), and left colon. Concurrently, it enabled us to precisely determine the positions of the AO, the two flexures, and the point at which the colonoscopy extends beyond the body (corresponding to the end of the rectum). Furthermore, we calculated the total inspection time by measuring the duration from the ATC to the point outside the body.

#### Appendix orifice time coordinated (ATC) layer

In this study, ATC denotes the initiation time of cecum detection through the AO detection model, providing a temporal reference in colonoscopy videos. The duration from ATC to the conclusion of the procedure is referred to as withdrawal time, a critical quality metric for colonoscopies. Prolonged withdrawal times, extending up to 10 min, have been linked to higher adenoma detection rates^37^. We compiled AO, semi-cecum, and non-cecum images from 100 videos. Clinicians meticulously reviewed and trained on 1580 cecum, 967 semi-cecum, and 1580 out-of-focus images. The dataset was divided into a 0.6:0.2:0.2 train: validation:test ratio. AO images showcased cecum features, excluding those resembling the ileocecal valve (ICV). Semi-cecum images depicted the terminal ileum and A-colon features, occasionally combined with unrelated content. Out-of-focus images extracted from colonoscopy videos served as ‘negative class’ images for discriminating intestinal walls from foreign substances. The average AO estimation time error was 6.31 s.

#### Flexure recognition layer

**Figure 2:**
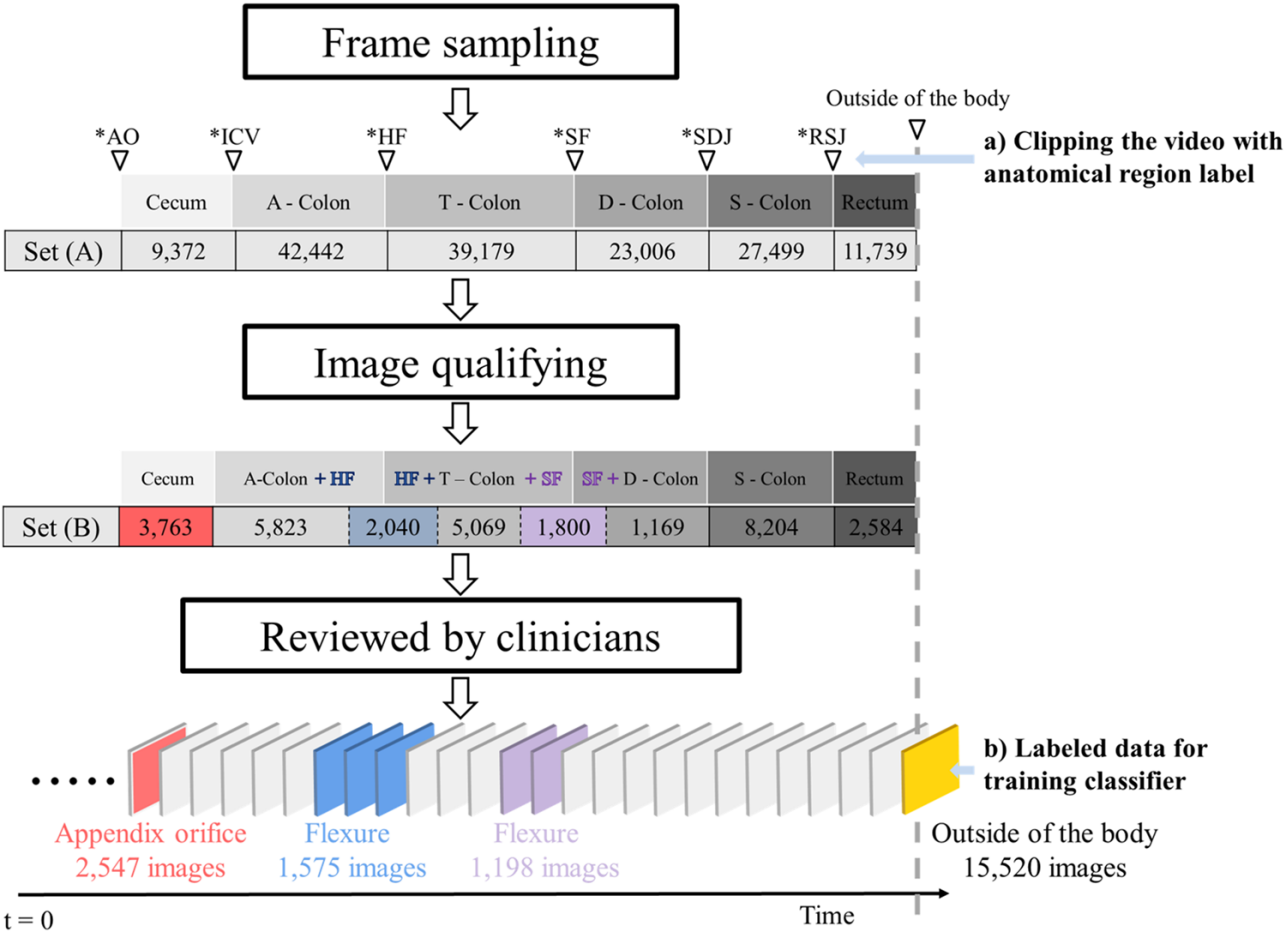
Training data acquisition process: Set (**A**): The number of images selected after frame sampling. Each anatomical segment is clipped using the timestamp as a reference. Set (**B**): The number of informative images listed according to the corresponding anatomical segment. After the candidate data is reviewed by clinicians, 2547 AO images, 2773 flexure images, and 15,520 outside-of-the-body images were obtained. (AO: appendiceal orifice, ICV: ileocecal valve, HF: hepatic flexure, SF: splenic flexure, SDJ: descending colon–sigmoid colon junction, RSJ: rectum–sigmoid colon junction, A-colon: ascending colon, T-colon: transverse colon, D-colon: descending colon).

Set (A) of Fig. 2 displays images of ‘candidate flexure,’ extracted from the point where the liver or pancreas appears blue through the mucosal wall, spanning from the first observed point to the last. Clinicians annotated timestamps in seconds near the flexure to serve as reference points (see Fig. 2a). This dataset encompassed flexure images interspersed with those taken before and after the flexure region. In total, we collected 3840 candidate flexure images. Clinicians meticulously reviewed these images to verify precise anatomical features, resulting in 2773 confirmed flexure images and 24,028 non-flexure images (depicted in Fig. 2b). Images from other segments were included in the "non-flexure" class to maintain a natural distribution, regardless of the imbalance in the number of images for each section (as depicted in Fig. 2). Non-flexure images were extracted in a ratio that preserved the relative composition across five sections: 434 from the cecum, 672 from the A-colon, 586 from the T-colon, 135 from the D-colon, and 946 from the sigmoid and rectum.

Recognizing flexures, which represent anatomical regions where the lumen bends, presents challenges, including instances where the camera loses focus in folded sections and encounters light reflections from nearby obstacles.

**Figure 3:**
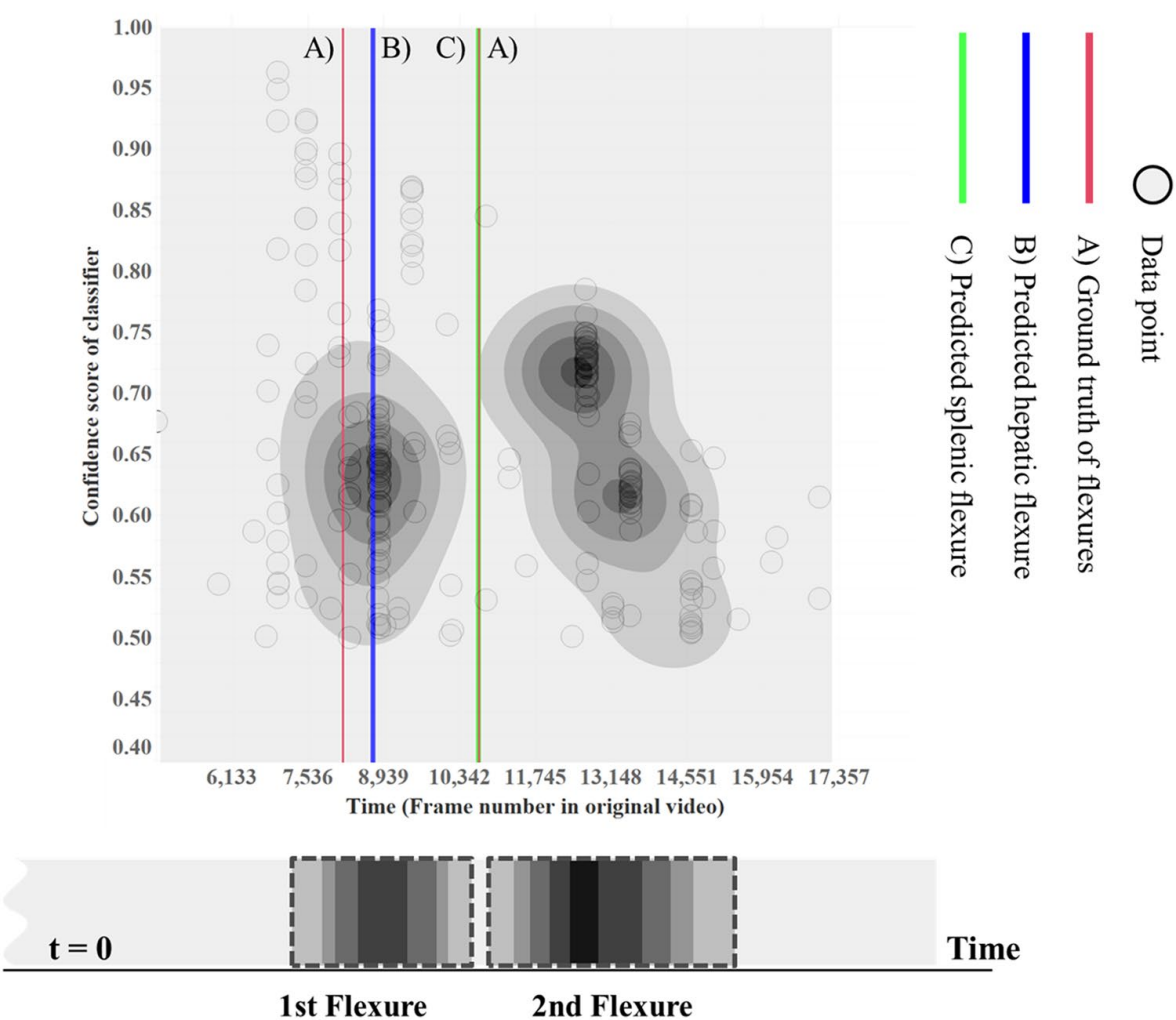
Summarization of colonoscopy video with density-based potential map (DPM): Flexure Recognition Layer. DBSCAN is applied to each frame in the video sequence with a high confidence score obtained by the flexure classifier. The result of the projection is the DPM at the bottom of the figure.

**Figure 4:**
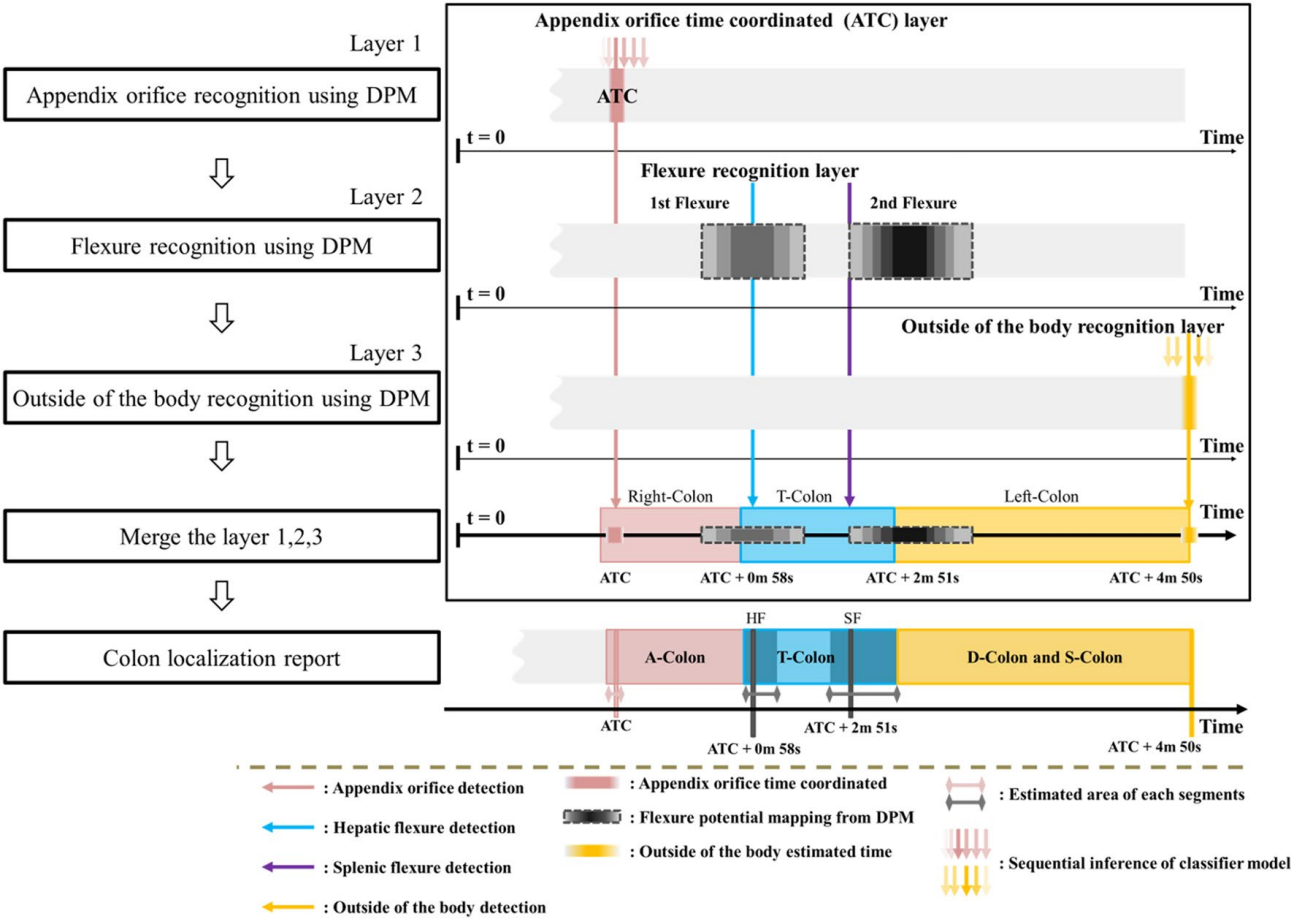
Analysis of anatomical sections using density clustering from three DPMs (Appendix Orifice, Flexures, and Outside the Body) for segment position estimation. Report sections: Right Colon (AO-HF), Middle Colon (T-Colon), and Left Colon (SF-outside of the body). DPM: Density-Based Probability Map.

To overcome this challenge, the flexure recognition layer treated out-of-focus and specular images as distinct classes. Using an informative frame filtering algorithm (see supplementary material A), we randomly selected 2773 out-of-focus and 2773 specularity images. Patient data were randomly divided to ensure that the same patient did not appear in different sets, resulting in a train: validation: test ratio of 0.6:0.2:0.2. In this study, the DPM analysis of the flexure layer was designed to recognize anatomical sections as probability intervals if they consistently appeared across temporally adjacent frames and clustered with sufficient frequency. The first and last clusters in the flexure layer were identified as representing HF and SF. Our observations revealed that the centroid of the first cluster matched the location of the HF, while the initial boundary of the last cluster corresponded to the SF. This suggests that accurate observation of the HF is feasible, as the subsequent section is the T-colon, allowing for relatively easy scope movement. However, in the case of the SF, as the scope advances into the D-colon and S-colon, the winding lumen causes the scope to face the colon wall, limiting visibility and posing challenges in obtaining informative images. In the DPM shown in Fig. 3, the high-confidence section is represented with a color gradient, particularly for the flexure. In density-based layer 2 (as shown in Fig. 4), a cross-section of the DPM for the flexure, obtained using the KDE method, is used as is (additional examples are provided in supplementary material Fig. C2).

#### Outside of the body recognition layer

This section outlines the methodology employed to identify the point at which the colonoscope exits the anus. Within the "outside of the body" recognition layer, images featuring characteristics of the external body surface were utilized to determine the conclusion of the inspection process. Specifically, the termination point from the SF to the external body boundary was defined as the left colon. The rectum could also be indirectly estimated as it represents the final part of the withdrawal phase. Additionally, we extracted frames captured after the anus from the training set videos to compile the training data for the "outside of the body" recognition.

Following this, an equal number of informative frames were extracted from the five colonoscopy segments, namely the cecum, A-colon, T-colon, D-colon, and S-colon, which collectively represent the "inside of the intestine." Similar to the models discussed in sections “Appendix orifice time coordinated layer” and “Flexure recognition layer”, the training set consisted of 9312 images, while the validation and test sets each comprised 3104 images, with a distribution ratio of 0.6:0.2:0.2.

#### Merging the density-based potential map layers

In Fig. 4, three layers of DPM collaborate to generate a prospective report, offering estimations of anatomical sections. This report serves a crucial role in aiding the recognition algorithm to identify the right, middle, and left colon, and concurrently estimate the rectum when the endoscope exits the body. Time calculations are rooted in the detection of the AO, with the detection time marked as ATC within the first layer. The potential report integrates information from layers 1, 2, and 3, taking into consideration the evaluation time errors associated with each estimated area. For instance, the HF model in layer 2 visualizes a time interval ranging from 8.63 s before the estimated evaluation time to 13.27 s after, indicating the probable HF appearance area. The ultimate output of this sophisticated recognition algorithm furnishes a detailed timeline, delineating the arrival times of the A-colon, T-colon, D-colon, and S-colon. This temporal mapping serves as a valuable tool for comprehending the chronological sequence of the colonoscopic examination.

### Ethics approval

Approval of all ethical and experimental procedures and protocols was granted by the institutional review board (IRB) in Seoul National University Hosipital (IRB No: H-2001-083-1095). Due to the retrospective nature of the study, H-2001-083-1095 waived the need of obtaining informed consent.

## Results

To address this, we determined an optimal cluster size. Given our utilization of time-series data, we set the epsilon value at 30 frames, which is equivalent to a 1-s window from the current frame for assessing region characteristics. The minimum sample size was determined through experimentation (please refer to supplementary material C). Subsequently, we applied the DBSCAN algorithm^33^ to the data presented in the anatomical segments report, resulting in the generation of a Density-Based Probability Map (DPM) (depicted in Fig. 3). In this figure, the blue line represents the estimation for the HF, while the purple line represents the estimation for the SF. The red vertical line denotes the ground truth timestamp provided by clinicians. Evaluation results for the AO, flexure, and outside-of-the-body classifiers are presented in Table 2.

**Table 2:** Results of the evaluation of the proposed inferencing model (using the modified AlexNet described in supplementary material B).

	Sensitivity (= Recall)	PPV (= Precision)	F1-score	NPV	Specificity	Accuracy
Cecum	0.8323	0.8680	0.8498	0.8337	0.9160	0.8463
Semi-cecum	0.6649	0.7633	0.7107	0.8676	0.9344	0.8463
Not-cecum	0.9715	0.8672	0.9164	0.8305	0.8929	0.8463
Flexure	0.8123	0.8911	0.8499	0.9267	0.9598	0.7960
Non-flexure	0.8159	0.6859	0.7453	0.9279	0.8637	0.7960
Outside of the body	0.9997	0.9997	0.9997	0.9997	0.9997	0.9997
Inside of the body	0.9997	0.9997	0.9997	0.9997	0.9997	0.9997

In the density-based analysis of the ATC layer, the AO classifier utilized frames from the cecum and semi-cecum to delineate the ATC region. Among the models proposed within the modified AlexNet (please refer to supplementary material B), the flexure classifier model exhibited the highest accuracy, with a sensitivity of 81.23% for flexure and 81.59% for non-flexure. Notably, the evaluation datasets from 20 videos were not included in the training dataset used for the classifier model.

In Fig. 3, the horizontal axis represents the frame index of a frame where the probability of each section exceeds 0.5, while the vertical axis represents the softmax probability.

### Comparison of classification CNN models

A performance comparison of image classification models for different classes, including EfficientNet^38^, Inception-v4^39^, AlexNet^32^, Squeeze-and-Excitation Network^40^, ResNet50^41^, and DenseNet^42^, was conducted. To ensure a fair comparison, consistent training conditions were maintained with 100 epochs, a learning rate of 1e-3, and the use of the Adam optimizer. Experiments were conducted in Python 3.7.6 using Keras version 2.4.3 and TensorFlow version 2.3.0. In cases of early stopping, training was halted if the validation loss for each epoch did not improve by 0.0005 or more for five consecutive deductions.

Additionally, potential performance differences based on image size were explored. Specifically, EfficientNet-B3 using 300 × 300 size images was compared with Inception-v4 using 299 × 299 size images. Among these models, AlexNet emerged as the top performer, attributed to its relatively smaller number of layers, preventing overfitting due to an excessive number of parameters^43^. The flexure classifier’s training dataset of 6660 images, constituting 60% of the total data, contributed to AlexNet’s superior performance (Detailed composition of the training data for each comparative model is extensively described in Supplementary Material B).

### Anatomical section recognition error

**Figure 5:**
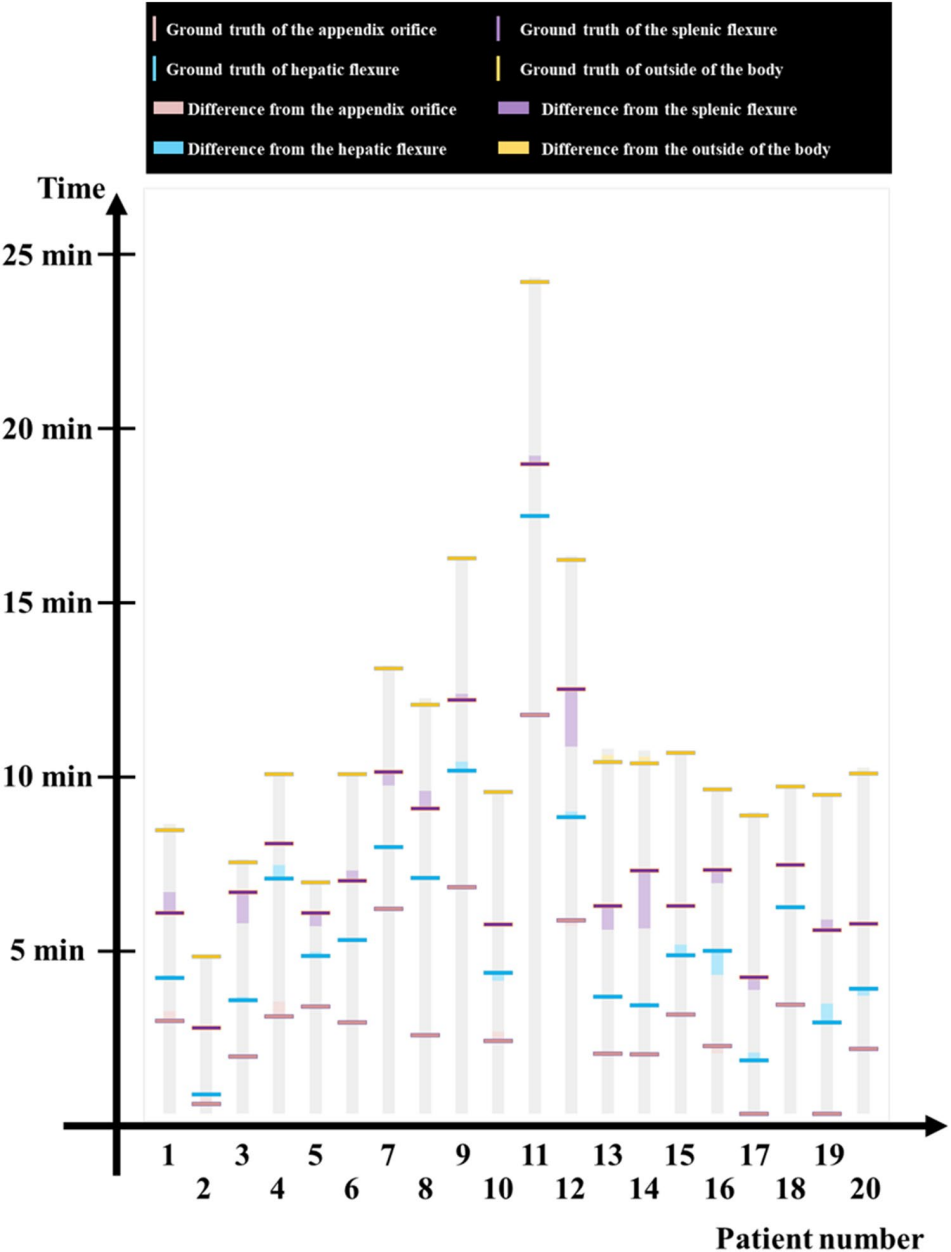
Difference in time estimation using DPM. The interference results of the proposed density clustering-based automatic reporting system can be confirmed through a horizontal bar. The test set time difference with ground truth is expressed for 20 patients. (Table C1 in supplementary material C).

The ATC layer and the flexure recognition layer collectively generate a result graph with probabilities to analyze densely clustered frames that yield high-confidence classification outcomes for the anatomical regions. Figure 5 illustrates the time discrepancies computed via the DPM. Based on the DPM analysis results, the predictions for AO, HF, and SF exhibited average errors of 6.31 s, 9.79 s, and 27.69 s, respectively. The disparity between the detection time and the correct answer time is expressed in seconds. For instance, taking case 17 in Fig. 5 as an example, it can be inferred that the ATC detector module identified AO 1.33 s later, while the flexure classifier identified HF 13.67 s after and SF 22 s ahead of the ground truth.

The error outcomes for the 20 test set videos were as follows: HF was detected with an average time error of 9.79 s, and SF was detected with a time error of 27.69 s. The outside-of-the-body model generally detected the end of the colonoscopy with minimal time error (3.26 s). As a result, there was no significant deviation between the model’s predictions and the ground truth.

## Discussion

In this paper, we presented a CNN-based image classification model that recognizes the anatomical section of the colon using frame-wise density intervals of colonoscopy videos; in this study, we used 100 colonoscopy videos. We presented a DPM to recognize the anatomical section of the colon in the colonoscope video. In addition, we introduced a DBSCAN-based analysis that visualizes anatomic fiducials as core points, proposes a spatial context, and localizes colon segments during a colonoscopy procedure. We chose GCF and the specularity masking method to handle non-informative images in colonoscope videos, which filtered blurry image frames and specular regions, respectively.

The developed model autonomously classifies the anatomical section of the colon into three parts. Furthermore, our proposed method opens the possibility of generating an infographic commentary for colonoscopy videos, providing visualization information that reflects the anatomical location and order of each segment. However, existing studies often involve a time-consuming and burdensome process, necessitating optical flow calculation solely for camera motion detection or the integration of additional sensors^21^. Approaches to determining the 6 Degrees of Freedom (6 DOF) pose of the camera involve estimating rotation and translation parameters, which can vary for each camera^22,23,44^. Additionally, correcting for disturbances, especially those caused by shaking, is imperative.

Furthermore, the value of the data in this study is significant, as it was trained using actual colonoscopy videos from 100 patients rather than a simulator. Simulators of the colon lack real-time peristaltic movement and do not undergo shape deformations, making them less representative of actual conditions^20,44,45^.

The key differences between our study from previous similar works are the following:

(i) The large intestine is divided into three sections without relying on the depth or pose parameter of the camera. The division utilizes only a computer vision-based segment location estimation, without the need for an external sensor.
(ii) The data required for all algorithm procedures can be obtained solely from simple colonoscopy videos.
(iii) The data are obtained from colon inspections of actual patients, not simulations, ensuring considerable accuracy and robustness of results.

### Contribution of anatomical segmentation

Accurate lesion localization within the colon is crucial for the effective detection of colonic abnormalities during colonoscopy^46,47^. Precisely determining the tumor location can offer valuable insights into clinical prognosis^48^ and treatment strategies^47^. Various methods, including medical imaging modalities such as magnetic resonance imaging (MRI)^49^, ultrasound^50^, and computed tomography (CT)^51^, have been employed to pinpoint lesion locations within the colon. However, challenges arise due to the colon’s deformable nature when measuring transformation parameters using external sensors. Some studies have aimed to identify colon segments through image processing with colonoscopes to capture previously overlooked areas^15,16^, while others have reconstructed spatial information during the withdrawal phase to guide the endoscope back to previously missed sections^43,52^. Unfortunately, these methods lack the utilization of anatomical landmarks or fiducials associated with specific colon sections^53,54^. The algorithm traditionally applied to simulators assumes an analysis based on a fixed structure^44,45^. Additionally, algorithms that return reconstruction as their output are impractical for real-world usage because they need to recalculate when the gastrointestinal tract moves^20^.

**Figure 6:**
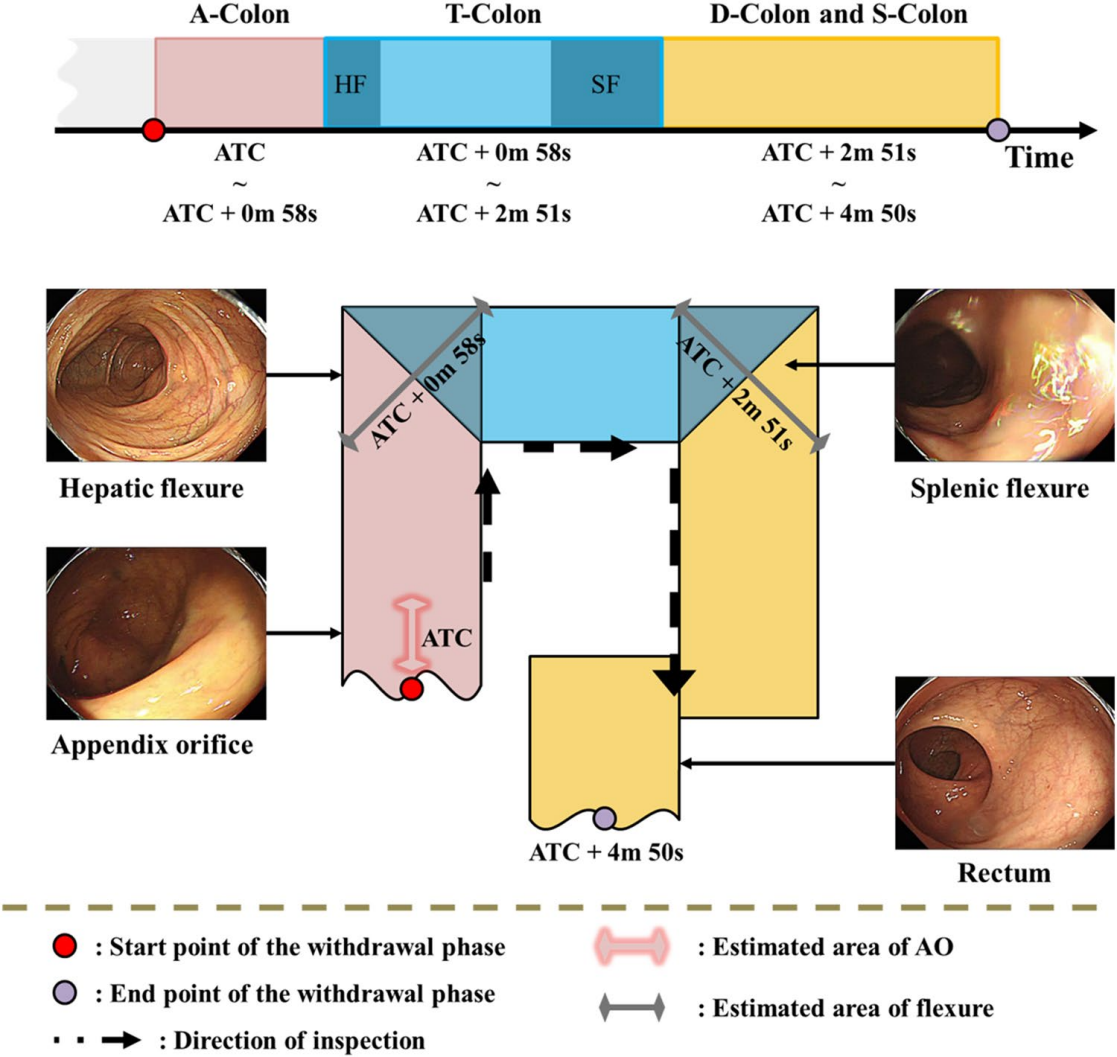
Reporting result of the proposed system. A-colon: ascending colon, T-colon: transverse colon, D-colon: descending colon, S-colon: sigmoid colon, ATC: appendix orifice time coordinated.

In contrast, our proposed algorithm uniquely identifies the position of corresponding segments in dynamic colon videos where peristaltic movements are continuous. It promptly addresses common issues in colonoscopy videos, such as motion blur and light reflections in frames, ensuring no computational delays in the model. Designed to distinguish segment locations within colonoscopic videos amid frequent peristaltic movements, our algorithm eliminates the need for additional sensors beyond the colonoscopy equipment. It sidesteps the complexities of parameter calculations and can be directly applied to actual patient videos, enhancing its practical utility. In our study, we classified the entire colon into right, middle, and left segments, utilizing information related to AO , flexures, and the external region (Fig. 6). The severity of colorectal polyps can vary depending on the examined region among the three areas (right colon, middle colon, and left colon). This approach provides clinical advantages by directly leveraging anatomical indicators, contrasting with traditional image-based colonoscope position estimation algorithms.

Unlike previous methods relying on motion for location and trajectory estimation, which necessitate the acquisition of translation transformation matrices and coordinate system rotations^22,23,44^, our study achieved high accuracy in segment classification within the large intestine without depending on camera intrinsic parameters^17^. Furthermore, our approach accommodates variable camera movement during colonoscopy, where the colonoscope may move backward for observation, revisit previous sections, or linger in specific areas for polyp removal. Consequently, the order and appearance of colon segments, including AO, flexures, T-Colon, D-Colon, S-Colon, and Rectum, may not follow a linear sequence and can overlap. Some segments may even reappear after initial identification. Nevertheless, our approach allows us to estimate the location of each colon segment, enabling the classification of the sequence of anatomical sections, even in cases of repeated sections.

### Visualization of density-based potential map

During the withdrawal phase of a colonoscopy, the colonoscope moves backward to exit the colon. Consequently, the field of view continuously observes areas that were previously traversed for a few to tens of seconds. Flexure images can still be observed even when the scope isn’t precisely positioned in that region. Therefore, our aim was to detect flexures as clusters rather than timestamps. In our testing, the colon localization algorithm provided detection times for AO, flexures, and the point outside the body in 20 colonoscopy videos. On average, the AO detection error was 6.31 s. In nine of these cases, AO was detected earlier than the correct timestamp, with an average lead time of 4.35 s. In cases where AO was detected later than the correct timestamp, an average delay of 7.92 s was recorded. For HF detection, the error averaged 9.79 s, while for SF detection, it was 27.69 s. The detection of the point outside the body, marking the end of the withdrawal phase, exhibited an average error of 3.26 s.

To plan our research and guide our segment detection patterns using DPM, considering the colon’s anatomical features, we conducted a focus group interview. We anticipated that the hepatic flexure region would become clearer during the T-colon phase. The DBSCAN analysis confirmed that the first core point in the DPM with the flexure classifier corresponded to the HF’s time index. Similarly, DPM with the ATC detector predicted AO as the first core point during colonoscopy. As the scope moved into the D-colon after the SF, it faced the mucosal wall, and we had to supply water due to limited movement in these segments. We expected the DPM’s foremost time index in the SF area to be closely related. In general, our predictions aligned with the DPM results, reflecting the colon’s anatomical structure.

### Limitations and future work

The limitation of this study is the challenge of performing external validation due to the absence of public data collected with the same protocol. Moreover, gastrointestinal clinicians demonstrated proficiency in distinguishing each anatomical region during data collection. In our subsequent study, we plan to collect prospective data from other institutions and apply this algorithm to establish its validity through external validation. Additionally, each training dataset for the developed models exhibited class-wise imbalances. While this is a common occurrence in medical data, we chose not to apply augmentation to address this issue in our study. The data used in this research was deemed valid for training only after consensus from multiple clinical experts, who identified the corresponding segments. Conversely, concerns were raised that augmented data, when viewed by clinical experts, might lack certainty in preserving valid information about anatomical landmarks’ features in colonoscopy videos. Furthermore, the model structure employed in this study was designed with three independently developed models, each specializing in learning and distinguishing different types of segment features. The inference results for each segment were then stacked to form the overall model (Figs. 1 and 4). This design was considered to mitigate the impact of data imbalance on the entire model, providing a potential solution.

In addition, attempts were made to separate the boundary between D-colon and S-colon and the anatomical section recognition to distinguish HF and SF into different sections. Accurately identifying the junction between the D-colon and S-colon in endoscopic videos of real patients has proven to be challenging, presenting difficulties for both image-based recognition models and gastroenterologists in the field. The delineation of D-colon and S-colon boundaries introduces complexities in training models for image analysis using deep learning. Despite challenges in detecting the SDJ, one avenue for addressing this issue involves leveraging the characteristics of folds. Folds in the D colon exhibit a rounded shape due to fixation to the posterior peritoneum, while those in the S colon may display a triangular shape influenced by the tenia coli^55^. We are exploring methods to utilize these features.

However, accurately creating the ground truth for the boundary line of the D-colon and S-colon is difficult. We are considering a method of learning the exact boundary through simulator images obtained from the manikin simultaneously. Our future research will involve video research techniques that learn not only from the still images but also from the sequence^56^. Nevertheless, when considering left colon classification based on the SF, accurately distinguishing between the D-colon and S-colon may not have a significant clinical impact in terms of therapeutic approaches, as discussed clinically^57^. Likewise, training was limited by dividing it into more detailed sections because the observable features were ambiguous to distinguish the junction between S-Colon and Rectum. Nevertheless, it was meaningful to classify it as left, middle, and right in terms of clinical significance.

Consequently, our system employs an analytical methodology for a density-based clustering map. This map analyzes the frame-wise inference results of the classification CNN model, automatically representing each section’s distribution in the colonoscopy video as a stochastic value. This capability allows for the separation of three anatomical regions, providing a visualized report. If this visualized report is integrated into the polyp detection algorithm, it becomes possible to generate a report that automatically suggests the location of the detected polyp. This AI-based colon section recognition during colonoscopy holds the potential to contribute to future advancements in AI-assisted automatic reporting, offering efficiency gains and standardization.

### Supplementary Information

Supplementary Information.

## Data Availability

The data generated and/or analyzed during the current study are available from the corresponding author on reasonable request.
