# Supplementary material for: Density clustering-based automatic anatomical section recognition in colonoscopy video using deep learning
Source: Sci Rep. 2024 Jan 9;14:872. doi: 10.1038/s41598-023-51056-6 (PMC10776865; doi:10.1038/s41598-023-51056-6)
Supplement: Supplementary file 1 — Supplementary Information. [file 41598_2023_51056_MOESM1_ESM.docx]

**Supplementary information**

**Density clustering-based automatic anatomical section recognition in colonoscopy video using deep learning**

Byeong Soo Kim^1, †^, Minwoo Cho^2,3,4, †^, Goh Eun Chung^5, †^, Jooyoung Lee^5^, Hae Yeon Kang^5^, Dan Yoon^1^, Woo Sang Cho^1^, Jung Chan Lee^6,7,8^, Jung Ho Bae^5, *^, Hyoun-Joong Kong^2,3,4,10, *^, and Sungwan Kim ^6,7,9, *^

^1^Interdisciplinary Program in Bioengineering, Graduate School, Seoul National University, Seoul, 08826, Korea

^2^Innovative Medical Technology Research Institute, Seoul National University Hospital, Seoul, 03080, Korea

^3^Department of Transdisciplinary Medicine, Seoul National University Hospital, Seoul, 03080, Korea

^4^Department of Medicine, Seoul National University College of Medicine, Seoul, 03080, Korea

^5^Department of Internal Medicine and Healthcare Research Institute, Healthcare System Gangnam Center, Seoul National University Hospital, Seoul, 06236, Korea

^6^Department of Biomedical Engineering, Seoul National University College of Medicine, Seoul, 03080, Korea

^7^Institute of Bioengineering, Seoul National University, Seoul, 08826, Republic of Korea

^8^Institute of Medical and Biological Engineering, Medical Research Center, Seoul National University, Seoul, 03080, Korea

^9^Artificial Intelligence Institute, Seoul National University, Research Park Building 942, 2 Fl., Seoul, 08826, Korea

^10^Medical Big Data Research Center, Seoul National University College of Medicine, Seoul, 03087, Korea

**^*^** bjh@snuh.org (J.H. Bae); [gongcop7@snu.ac.kr (H.-J](mailto:gongcop7@snu.ac.kr%20(H.-J). Kong); [sungwan@snu.ac.kr](mailto:sungwan@snu.ac.kr) (S. Kim)

**^†^** Byeong Soo Kim, Minwoo Cho and Goh Eun Chung contributed equally to this work (co-first author).

**Correspondence to:**

**Sungwan Kim**

Department of Biomedical Engineering, Seoul National University College of Medicine, 101 Daehak-ro, Jongno-gu, Seoul, 03080, Korea

Tel: +82-2-2072-3126

Fax: +82-2-745-7870

E-mail: sungwan@snu.ac.kr

ORCID: 0000-0002-9318-849X

**Hyoun-Joong Kong**

Department of Medicine, Seoul National University College of Medicine, 103 Daehak-ro, Jongno-gu, Seoul, 03080, Republic of Korea

Transdisciplinary Department of Medicine & Advanced Technology, Seoul National University Hospital, 101, Daehak-ro Jongno-gu, Seoul, 03080, Republic of Korea

Tel: +82-2-2072-4492

Fax: +82-504-446-1012

E-mail: [gongcop7@snu.ac.kr](mailto:gongcop7@snu.ac.kr)

ORCID: 0000-0001-5456-4862

**Jung Ho Bae**

Department of Internal Medicine and Healthcare Research Institute, Healthcare System Gangnam Center, Seoul National University Hospital, 152 teheran-ro, Gangnam-gu,Seoul, 06236, Republic of Korea

E-mail: bjh@snuh.org

ORCID: 0000-0001-7669-1213

**Supplementary material A**

**Noninformative frame filtering: global contrast factor and specular reflection detection method**

“Out-of-focus” images refer to cases where the scope is too close to the mucosal wall, when it is rushing (motion artifact), and when a foreign substance covers the camera lens. The luminance distribution of these images was monotonous compared to that of other images with the same resolution. We used global contrast factor (GCF) as a feature detector to filter out the out-of-focus images.

GCF is obtained by normalizing the pixel value $k$, where $k\in\left\{ 0, 1, \ldots, 254, 255 \right\}$, by dividing it by 255. Perceptual luminance is obtained by multiplying the gamma ($\gamma$ = 2.2) value for luminance correction.

$linear luminance: l=\left( \frac{k}{255} \right)\gamma$. (A.1)

$Perceptual luminance: L=100* \sqrt{l}$. (A.2)

Thereafter, the average of the perceptual luminance values on the upwards, downwards, left, and right sides of the pixels are quantified. Considering that image has the dimension of $w$ pixel width and $h$ pixels height, we get:

$lc_{i}= \frac{\left| L_{i}- L_{i-1} \right|+\left| L_{i}- L_{i+1} \right|+\left| L_{i}- L_{i-w} \right|+\left| L_{i}- L_{i+w} \right|}{4}$, (A.3)

$C_{i}= \frac{1}{w*h}*\sum_{i=1}^{w*h} lc_{i}$, (A.4)

where $C_{i}$ represents the average local contrast current resolution. $C_{i}$ is repeatedly calculated for nine super-pixels. Super-pixel is an image of a new resolution obtained by pooling 1, 2, 4, 8, 16, 25, 50, 100, and 200 pixels into one pixel.

Regression equation: $w_{i}$ = (-0.406385 * $\frac{i}{9}$ + 0.334573) * $\frac{i}{9}$ + 0.0877526, (A.5)

$GCF= \sum_{i=1}^{N} w_{i}*C_{i}$. (A.6)

GCF is the sum of the values from $C_{1}$ to $C_{9}$ multiplied by the regression equation $w_{i}$; $w_{i}$ is obtained from an experiment that approximates the contrast similar to that felt by humans. To make an operational definition of the noninformative image, we extracted 500 images where it was difficult to determine whether the image is informative due to the appearance of motion blur and artifact (Ali et al., 2019). As a result, the average GCF value for the 500 images was 3.446 ± 0.758, and 95% of images had a GCF value less than 4.6. Therefore, a GCF value of 4.6, including all experimental out-of-focus data groups except the 5%, was set as the threshold for the out-of-focus images.

Specularity refers to cases where the saturation and intensity of light reflection in an image corresponds to an outlier value. When there is a structural protrusion; such as polyps, feces, or foreign body; or when the protrusion is too close to the light source, the light from the colonoscope is reflected in one direction (Tchoulack et al., 2008). This reflection hinders the image from being recognized, and is called specularity or specular reflection. We used the specular reflection detection method to filter such images.

The intensity $k$ and saturation $s$ of specularity are respectively built as follows:

$k=\frac{1}{3}*\left( r+g+b \right)$, (A.7)

$s= \left\{ \begin{aligned} \frac{1}{2}*\left( 2r-g-b \right)=\frac{3}{2}*\left( r-m \right), if(b+r)\geq2g \\ \frac{1}{2}*\left( r+g-2b \right)=\frac{3}{2}*\left( m-b \right), if\left( b+r \right)<2g \end{aligned} \right.$ , (A.8)

where r, g, and b represent the red, green, and blue components of the image, respectively.

A pixel $p$ could be a part of the specular region if it meets the following conditions:

$k_{p}\geq\frac{1}{2}*k_{max}$, (A.9)

$s_{p}\geq\frac{1}{3}*s_{max}$. (A.10)

The specular mask refers to the area where the pixel intensity is more than half of the maximum intensity of the intensity map of the image, and where the pixel saturation is more than a third of the maximum saturation of the saturation map of the image (Tchoulack et al., 2008). A pixel surrounded by pixels satisfying the specularity condition is considered as a specular region as well. When a pixel satisfying the specularity condition exists within the 7 × 7 sliding window, the central pixel is included in the specularity mask. The noninformative frames were filtered by calculating the area of the specularity mask thus created relative to the total image size.

To determine the specularity threshold through experimental definition, we manually selected 350 images that were difficult to analyze due to specular reflection, and their relative size was calculated as a percentage. As a result, images with a $\frac{Specularity mask size}{Image size}$ of less than 4% were considered informative frames even if the specular reflection was found through histogram analysis.

**Supplementary material B**

**Comparison of CNN models for the ATC detector module, flexure classification model, and outside of the body classification model**

In this study, we compared the accuracy of modified AlexNet (Krizhevsky et al., 2012), ResNet50 (He et al., 2016), Squeeze-and-Excitation network (Hu et al., 2018), DenseNet (Huang et al., 2017), Inception-v4 (Szegedy et al., 2017), and EfficientNet-B0 and EfficientNet-B3 (Tan and Lee, 2019). Among these, the modified AlexNet exhibited the highest accuracy.

Typically, modified AlexNet utilizes the AlexNet architecture of using five convolution layers and three dense layers; however, it performed batch-normalization in front of the activation function of all convolution layers. We used Swish instead of ReLU as the activation function, except for the softmax function of the last fully connected layer that returns a classification result. In addition, a total of nine layers of architecture were used by adding one more dense layer in the AlexNet, following three dense layers after the five convolution layers, to improve its performance. In this case, the sixth dense layer has 8,192 nodes, and the seventh and eighth layers use 4,096 nodes as they are. Considering this complexity, we selected an activation function that can propagate the total activated value to the next layer. This model exhibited better accuracy with Swish compared to ReLU.

**Supplementary material C**

**Density-based spatial clustering of applications with noise (DBSCAN) for DPM**

DBSCAN is a clustering algorithm in which data points are closely concentrated; therefore, it clusters high-density parts. If a point is above the minimum points (MinPts) within a radius epsilon (eps) from a point, it is recognized as a cluster. To apply DBSCAN, the appropriate parameters “eps” (epsilon) and “MinPts” (minimum number of points of each cluster) should be known. A point that satisfies the conditions of this cluster is called a core point. If another core point is included within the eps radius of the corresponding point for all points, we group these core points into one cluster. However, there may be outliers that do not belong to any cluster; the DBSCAN algorithm separates such data, groups them as noise points, and excludes them from the cluster. As a result, a noise point is not displayed on DPM.

DBSCAN fixes the eps radius at 30 because if a total of 30 frames, back and forth, are set at eps, the data within 1 s can be formed as core points. This means that if flexure is observed continuously for more than 2 s, it expands in seconds, and is determined as flexure interval and drawn on the DPM.

The MinPts value for forming a cluster is 10% of the total number of points, and it does not exceed 30. Since the flexure segment must appear twice in the video, the DBSCAN result was made into a kernel density estimation (KDE) graph to estimate the data distribution. Fig. C.1 displays the graphs created to find the appropriate MinPts applied to the AO classification model. As seen in the figure, a graph was drawn with the epsilon fixed at 30, and the number of MinPts were increased to the maximum possible epsilon value of 60 to determine the appropriate MinPts in DBSCAN. It can be seen that during each video analysis, the smallest MinPts value, with no change in the core point of the cluster despite the increase in MinPts, was designated as the representative MinPts value. Video Nos. 1–4 in Fig. C.1 were set to appropriate MinPts of 21, 25, 15, and 16, respectively; this forms a DPM. KDE was graphically represented by considering all of the probability density functions specified among the various possibilities of data. Our study was used to estimate the characteristics of the original flexure segment from the anatomical segments report drawn by the flexure classifier. In conclusion, the time stamp of the three colon segments was obtained by classifying the time between HF and SF as T-colon, which is the middle colon. An essential consideration in interpreting DPM is finding the centroid of the cluster that appears first in chronological order among visible clusters.

**
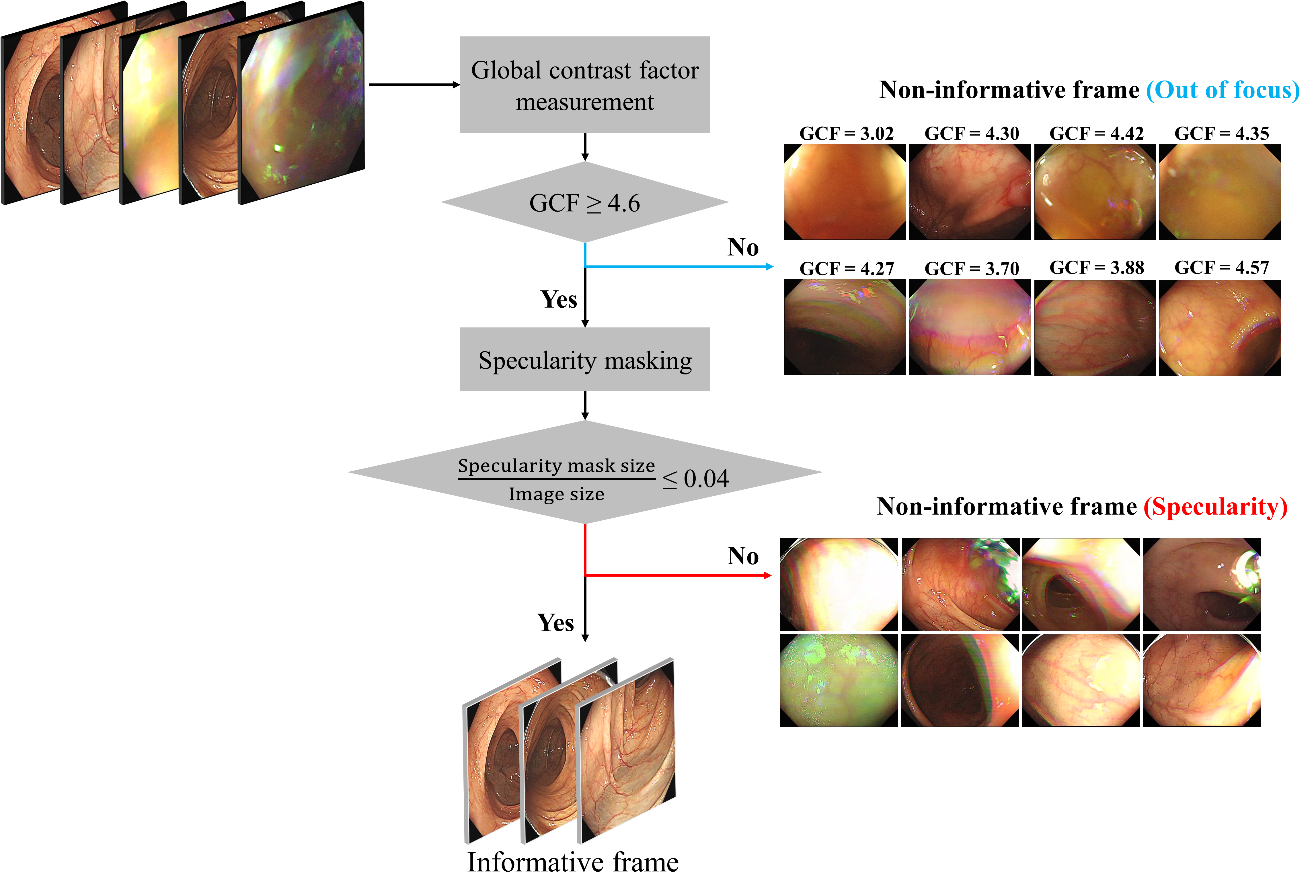
**

**Supplementary Figure A1.** Noninformative frame filtering. GCF: global contrast factor.

**Supplementary Table B1.** Classification performance, accuracy, and trained epoch of various models

| ATC detector module | | Accuracy (%) | Input image size  (Width pixel x Height pixel) | Training epochs  (using early stopping) | Model | Accuracy (%) | Input image size  (Width pixel x Height pixel) | Training epochs  (using early stopping) |
| --- | --- | --- | --- | --- | --- | --- | --- | --- |
| *EfficientNet-B3* | | 67.070 | 300 x 300 | 27 | *EfficientNet-B0* | 66.828 | 224 x 224 | 14 |
| *Inception-v4* | | 68.281 | 299 x 299 | 17 | *SE-ResNet-50* | 51.211 | 224 x 224 | 9 |
| *Alexnet* | | 80.751 | 227 x 227 | 50 | *ResNet50* | 61.380 | 224 x 224 | 17 |
| **Modified *Alexnet*** | | **84.625** | **227 x 227** | **86** | *DenseNet-264* | 68.523 | 224 x 224 | 16 |
|  | |  |  |  |  |  |  |  |
| Flexure classification model | | Accuracy (%) | Input image size  (Width pixel x Height pixel) | Training epochs  (using early stopping) | Model | Accuracy (%) | Input image size  (Width pixel x Height pixel) | Training epochs  (using early stopping) |
| *EfficientNet-B3* | | 59.838 | 300 x 300 | 24 | *EfficientNet-B0* | 56.408 | 224 x 224 | 19 |
| *Inception-v4* | | 56.859 | 299 x 299 | 21 | *SE-ResNet-50* | 48.556 | 224 x 224 | 15 |
| *Alexnet* | | 74.007 | 227 x 227 | 46 | *ResNet50* | 46.119 | 224 x 224 | 12 |
| **Modified *Alexnet*** | | **79.603** | **227 x 227** | **96** | *DenseNet-264* | 49.594 | 224 x 224 | 21 |
|  | |  |  |  |  |  |  |  |
| Outside of the body classification model | | Accuracy (%) | Input image size  (Width pixel x Height pixel) | Training epochs  (using early stopping) | Model | Accuracy (%) | Input image size  (Width pixel x Height pixel) | Training epochs  (using early stopping) |
| *EfficientNet-B3* | | 99.774 | 300 x 300 | 22 | *EfficientNet-B0* | 99.066 | 224 x 224 | 7 |
| *Inception-v4* | | 99.340 | 299 x 299 | 18 | *SE-ResNet-50* | 99.340 | 224 x 224 | 25 |
| *Alexnet* | | 99.952 | 227 x 227 | 24 | *ResNet50* | 99.259 | 224 x 224 | 26 |
| **Modified *Alexnet*** | | **99.968** | **227 x 227** | **17** | *DenseNet-264* | 50.000 | 224 x 224 | 6 |
|  |  | | | | | | | |

**Supplementary Table B2.** Appendix orifice time coordinated (ATC) detector model evaluation results (using the modified AlexNet)

|  | PPV  (= Precision) | Sensitivity  (= Recall) | NPV | Specificity | Accuracy |
| --- | --- | --- | --- | --- | --- |
| Cecum | 0.8680 | 0.8323 | 0.8337 | 0.9160 | 0.8463 |
| Semi-cecum | 0.7633 | 0.6649 | 0.8676 | 0.9344 |  |
| Not-cecum | 0.8672 | 0.9715 | 0.8305 | 0.8929 |  |
| *PPV: Positive predictive value  *NPV: Negative predictive value  *Confidence threshold: 0.5 | | | | | |

**Supplementary Table B3.** Flexure classification model evaluation results (using the modified AlexNet)

|  | PPV*  (= Precision) | Sensitivity  (= Recall) | NPV* | Specificity | Accuracy |
| --- | --- | --- | --- | --- | --- |
| Flexure | 0.8911 | 0.8123 | 0.9267 | 0.9598 | 0.7960 |
| Non-flexure | 0.6859 | 0.8159 | 0.9279 | 0.8637 |  |
| Out-of-focus | 0.8193 | 0.9332 | 0.9712 | 0.9256 |  |
| Specularity | 0.8195 | 0.6227 | 0.8716 | 0.9492 |  |

**Supplementary Table B4.** Outside of the body classification model evaluation results (using the modified AlexNet)

|  | PPV  (= Precision) | Sensitivity  (= Recall) | NPV | Specificity | Accuracy |
| --- | --- | --- | --- | --- | --- |
| Outside of the body | 0.9997 | 0.9997 | 0.9997 | 0.9997 | 0.9997 |
| Inside of the body | 0.9997 | 0.9997 | 0.9997 | 0.9997 |  |
|  | | | | | |


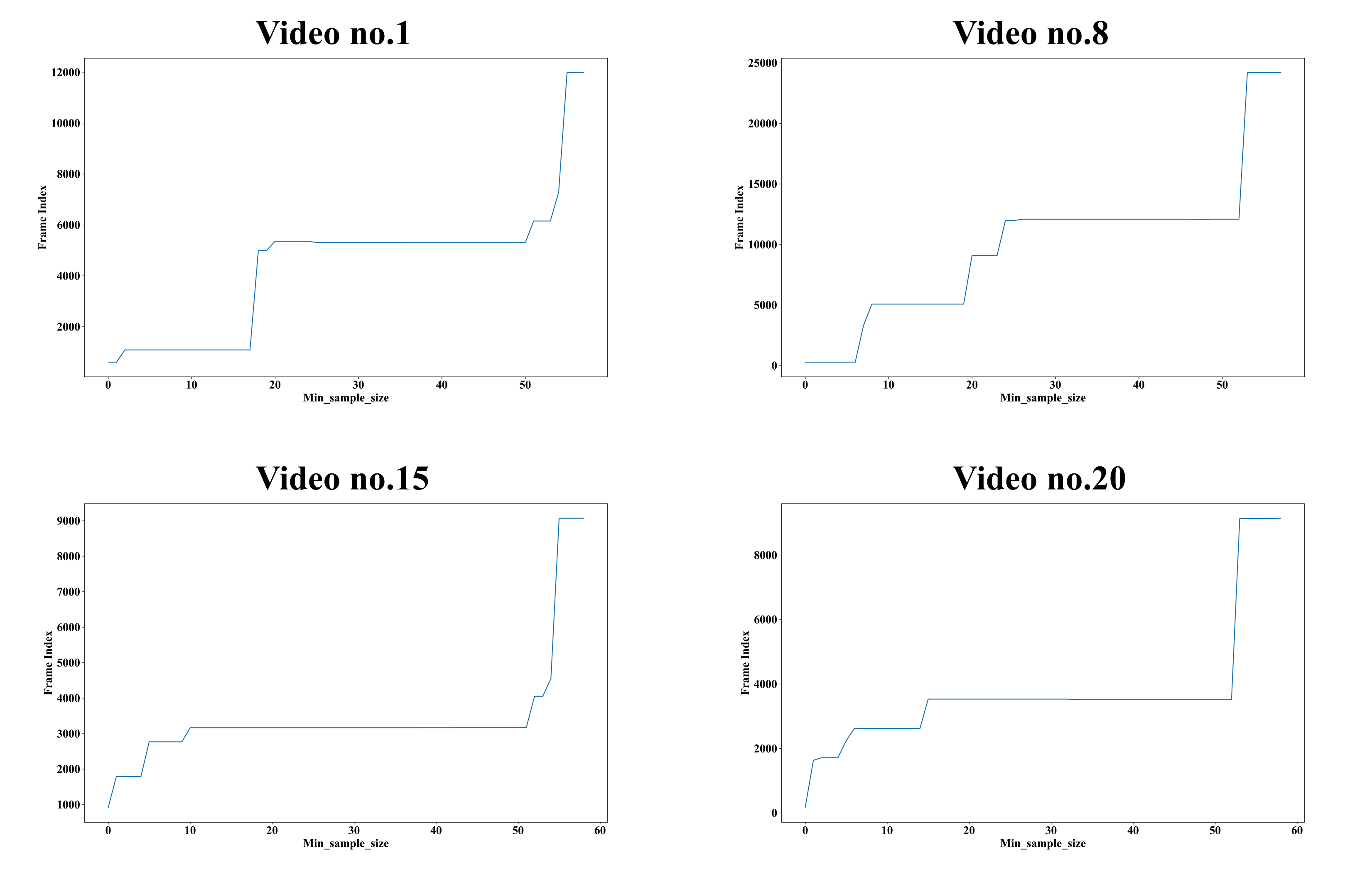


**Supplementary Figure C1.** Determination of appropriate MinPts value in the graph for the decision graph interpretation of the AO classification model.


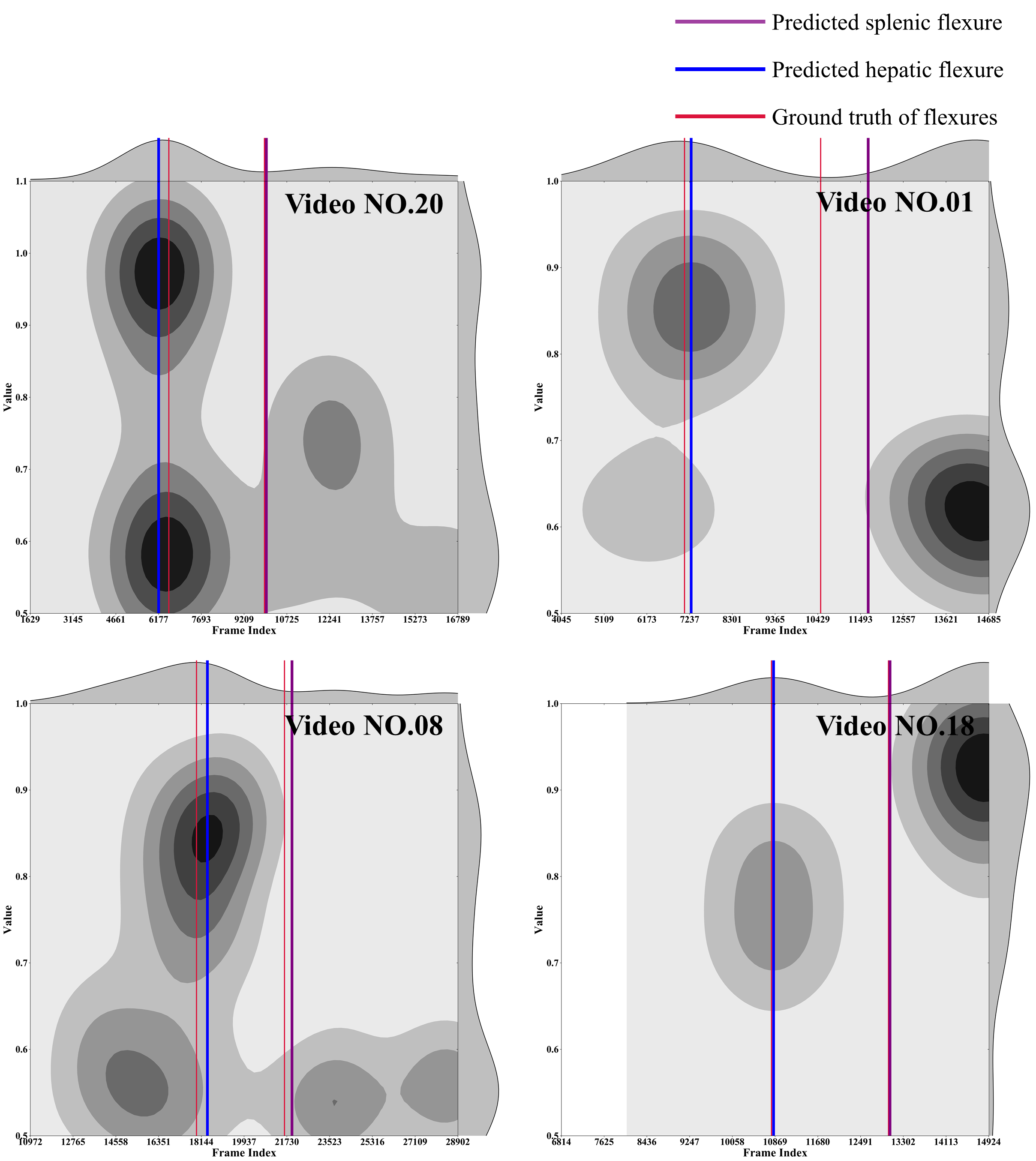


**Supplementary Figure C2**. DPM fraph examples for flexure.

**Supplementary Table C1.** Results of the proposed system using DPM

|  | **Ground truth** | | | | **Total video length** | **Difference in the second (m = minute, s = second)** | | | |
| --- | --- | --- | --- | --- | --- | --- | --- | --- | --- |
| Video  no. | Appendix orifice | Hepatic flexure | Splenic flexure | Outside of the body |  | Appendix orifice | Hepatic flexure | Splenic flexure | Out of the body |
| 1 | 2m 42s | 3m 57s | 5m 50s | 8m 14s | 8m 25s | 16.53s | 5.67s | 43.33s | 5.77s |
| 2 | 0m 17s | 0m 34s | 2m 30s | 4m 34s | 4m 37s | 10.03s | 0.33s | 2.33s | 1.10s |
| 3 | 1m 40s | 3m 18s | 6m 26s | 7m 18s | 7m 24s | -4.40s | 5.33s | -54.33s | 3.10s |
| 4 | 2m 50s | 6m 50s | 7m 51s | 9m 52s | 9m 56s | 25.17s | 23.33s | 4.00s | 1.57s |
| 5 | 3m 07s | 4m 35s | 5m 50s | 6m 43s | 6m 46s | -1.70s | 7.00s | -23.33s | 1.70s |
| 6 | 2m 39s | 5m 03s | 6m 46s | 9m 52s | 9m 55s | 5.50s | 2.17s | 18.00s | 1.37s |
| 7 | 5m 57s | 7m 45s | 9m 56s | 12m 56s | 13m 01s | 2.13s | 1.67s | -24.00s | 2.67s |
| 8 | 2m 17s | 6m 51s | 8m 52s | 11m 53s | 12m 04 | -5.17s | -1.00s | 30.67s | 5.50s |
| 9 | 6m 35s | 9m 58s | 12m 01s | 16m 08s | 16m 10s | 3.90s | 15.33s | 10.50s | 0.97s |
| 10 | 2m 07s | 4m 06s | 5m 30s | 9m 21s | 9m 23s | 16.27s | -8.67s | -10.00s | 0.93s |
| 11 | 11m 35s | 17m 22s | 18m 52s | 24m 10s | 24m 17s | 0.13s | 1.33s | 4.67s | 3.37s |
| 12 | 5m 37s | 8m 37s | 12m 20s | 16m 05s | 16m 12s | -9.20s | 0.67s | -98.33s | 3.37s |
| 13 | 1m 45s | 3m 24s | 6m 02s | 10m 13s | 10m 36s | -2.83s | -4.00s | -41.33s | 11.40s |
| 14 | 1m 44s | 3m 09s | 7m 04s | 10m 11s | 10m 33s | -0.63s | 0.00s | -110.67s | 10.80s |
| 15 | 2m 53s | 4m 36s | 6m 02s | 10m 29s | 10m 32s | -2.37s | 19.00s | 4.67s | 1.20s |
| 16 | 1m 58s | 4m 44s | 7m 05s | 9m 25s | 9m 28s | -12.47s | -42.33s | -23.33s | 1.37s |
| 17 | 0m 0s | 1m 33s | 3m 58s | 8m 40s | 8m 46s | 1.33s | 13.67s | -22.00s | 3.10s |
| 18 | 3m 10s | 6m 00s | 7m 14s | 9m 30s | 9m 31s | -0.37s | 1.33s | 6.00s | 0.43s |
| 19 | 0m 0s | 2m 39s | 5m 20s | 9m 16s | 9m 17s | 1.53s | 32.67s | 20.00s | 0.63s |
| 20 | 1m 53s | 3m 38s | 5m 31s | 9m 53s | 10m 03s | 4.63s | -10.33s | 2.33s | 4.77s |
|  |  |  |  |  | Mean | 6.31s | 9.79s | 27.69s | 3.26s |
